# Effects of 3D Scans on Veterinary Students’ Learning Outcomes Compared to Traditional 2D Images in Anatomy Classes

**DOI:** 10.3390/ani14152171

**Published:** 2024-07-25

**Authors:** Rebecca Schirone, Giuliano Mario Corte, Jan P. Ehlers, Christina Herre, Maximiliane Schmedding, Roswitha Merle, Joëlle Pachtmann, Mahtab Bahramsoltani

**Affiliations:** 1 Institute of Veterinary Anatomy, School of Veterinary Medicine, Freie Universität Berlin, Koserstraße 20, 14195 Berlin, Germany; 2 Institute of Veterinary Anatomy, Vetsuisse Faculty, University of Zurich (UZH), Winterthurerstrasse 260, 8057 Zurich, Switzerland; 3 Didactics and Educational Research in Health Science, Faculty of Health, Witten/Herdecke University, Alfred-Herrhausen-Straße 50, 58455 Witten, Germany; jan.ehlers@uni-wh.de; 4 ISME Bern and Avenches, Vetsuisse Faculty, University of Bern, Hochschulstrasse 6, 3012 Bern, Switzerland; 5 Institute of Veterinary Epidemiology and Biostatistics, School of Veterinary Medicine, Freie Universität Berlin, Königsweg 67, 14163 Berlin, Germany

**Keywords:** veterinary didactics, veterinary education, digital 3D models, 3D scans, 2D vs. 3D

## Abstract

**Simple Summary:**

3D models are increasingly popular in veterinary teaching. However, students struggle with transferring 2D textbook images to 3D anatomical structures. Therefore, this study aimed to compare the learning outcome of 3D scans and 2D images of horse and pig skulls. Furthermore, the relationship between spatial ability and learning outcome was analyzed when using the different learning materials. Second-year veterinary medicine students participated in a practical anatomy course using 3D scans or 2D images as learning material and performed a test to determine their spatial ability. Independently of the learning material, all groups displayed high learning outcomes with no significant differences between the groups. In addition, it could not be stated that 3D scans support students with lower spatial ability. Nevertheless, students preferred 3D scans, finding them more motivating, although they did not necessarily improve learning outcomes.

**Abstract:**

Students often struggle with interpreting traditional textbook images and translating them to anatomical structures. This study aimed to compare the impact of 3D scans versus 2D images on students’ learning outcomes when learning anatomical structures on skulls from horses and pigs. Furthermore, the correlation between spatial ability and learning outcomes using 3D scans or 2D images was examined. Second-year veterinary medicine students either used 3D scans or 2D images, annotated with arrows or numbers as learning material. Students’ anatomical knowledge was tested before and after the learning session, and spatial ability was assessed using the mental rotation test. All groups improved significantly in the post-test. However, the differences between groups were not significant, suggesting that 3D scans do not necessarily lead to higher learning outcomes. The analysis of the correlation between spatial ability and learning outcomes did not prove that students with weaker spatial ability benefit from 3D scans. Students preferred 3D scans over 2D images despite similar outcomes, suggesting they are valuable for learning. However, results show that the introduction of novel learning materials likely amplified the impact of reduced learning time on the 3D group, as these materials necessitated additional time for effective comprehension and integration.

## 1. Introduction

Anatomy represents one of the fundamental subjects encountered by students of veterinary and human medicine at the beginning of their studies. It forms an essential building block for further medical disciplines. By gaining knowledge and learning practical skills about anatomical structures, students acquire a foundation for related subjects, e.g., surgery, pathology, and radiology [1,2,3]. Moreover, anatomy holds substantial importance in the educational journey of medical students, influencing their learning experience both intellectually and emotionally [4]. Furthermore, expertise in anatomy is indispensable for graduate doctors and veterinarians [5,6,7]. Veterinarians of small animal practice underlined the importance of the ongoing process of learning and refreshing veterinary anatomy in order to competently carry out their profession. They emphasize the importance of teaching anatomy in the veterinary medicine curriculum. Additionally, the connection between the application of anatomy in their daily routine, psychomotor abilities, and professional skills was pointed out [6]. A study that was carried out with horses and farm animals showed that a solid and fundamental understanding of clinically relevant anatomy in combination with skills of recognizing complicated anatomical details and exploring unfamiliar areas, is crucial for the profession of a clinician. Adequate knowledge of veterinary anatomy seems to be essential for effective communication, enhancing trust and confidence for the competent performance of clinical tasks [7]. 

For this reason, the medical and veterinarian curriculum includes practical courses, which are carried out as cadaveric dissection [8,9]. They provide hands-on experiences that complement theoretical knowledge. The courses are indispensable for understanding the three-dimensional body, developing the ability to experience physical contact, understanding the topographical position of anatomical structures and organs and ultimately being able to master and interpret images derived from imaging procedures such as X-rays, ultrasound, or computer tomography [7,9,10]. The teaching materials used to prepare and support dissection courses have been predominantly two-dimensional drawings, supplemented by photographs and, more recently, videos. Visual materials provide valuable insights [11,12]. 

However, it is necessary to comment on the limitations of a two-dimensional surface. In practical anatomy courses, it has been shown that the transfer of information from two-dimensional (2D) teaching materials to the three-dimensional (3D) body was considered difficult by students [13,14]. The individual’s spatial ability displays a major influencing factor. Several studies explored the correlation between spatial ability and anatomy learning [15,16,17]. To gain a thorough knowledge of anatomy, one must understand the comprehensive structure of organs both individually and in relation to the entire body. Anatomical structures have their own functional specificity and are often shaped asymmetrically and irregularly. Mental processing and retention of 3D configurations and the understanding of their spatial relationships are essential components for performing clinical analyses and interpretations [18]. A recent study stated that students with high spatial ability skills are significantly superior to individuals with low spatial ability skills when learning with static 2D images instead of 3D animated models. Researchers suggested that people with weaker spatial imagination could learn significantly better with 3D models [19]. 

In the present study, the use of 3D scans compared to 2D images as learning material in anatomy classes was investigated. The following hypotheses were tested:

1. When learning the anatomical structures of the skull, students who use 3D scans as learning material have a higher learning outcome than students who use 2D images as learning material.
2. There is an interaction effect between spatial ability and the learning material used, with students with low spatial ability having a higher learning outcome with the use of 3D scans than with 2D images.

## 2. Participants, Materials and Methods

### 2.1. Participants

For the current study, data was raised during the practical comparative anatomy lesson on the skull for second-year veterinary medicine students in October 2022 (study 1) and October 2023 (study 2). 

During the previous anatomy lesson, a week before, the students were informed about the objectives and procedure of the study verbally and using an information letter. The students who agreed to take part in the study gave their informed consent for inclusion before they participated in the study by filling out and signing a data protection declaration. Students were assured that their involvement was voluntary, with data collection being anonymous. Participants were given the freedom to withdraw from the study at any time point without facing consequences. In addition, the participants were requested not to prepare for the upcoming lesson on the skull. The information letter and data protection declaration are provided in Supplement S1. 

### 2.2. Learning Materials

#### 2.2.1. Skulls 

In both study 1 and study 2, 32 horse skulls and 32 pig skulls were used in total. The used skulls belong to the anatomical collection of the Institute of Veterinary Anatomy at Freie Universität Berlin. 

#### 2.2.2. 3D Scans

3D scans of the horse and pig skulls, which were employed in this study, were generated using the handheld 3D scanner Artec Space Spider (Artec 3D, Luxembourg). It is a high-resolution scanner based on blue light technology. It is capable of capturing the geometry and texture of an object simultaneously by taking multiple pictures. To assemble and edit the finalized 3D model, the manufacturer software Artec Studio 16 (Artec 3D, Luxembourg) was used. 

First, the scanned background and interfering objects that do not belong to the model were removed via the “Erase” function, before scanning was continued. As the scanner was taking more scans simultaneously, two scans at once can be merged using the “Alignment” tool. To create one 3D object from the scans, the “Global registration” task was carried out to convert all-frame surfaces to a single coordinated system. The following “Sharp fusion” task melted and solidified the frames to create the 3D body. If the 3D body displayed an incomplete surface after the initial scans, either further scans were made to obtain more raw data or AI was used to generate a complete surface with the function “Fill holes”. After the 3D body was satisfactory, surface texture was provided via the “Texture” item. Raw data can be found in Supplement S5.

**Figure 1:**
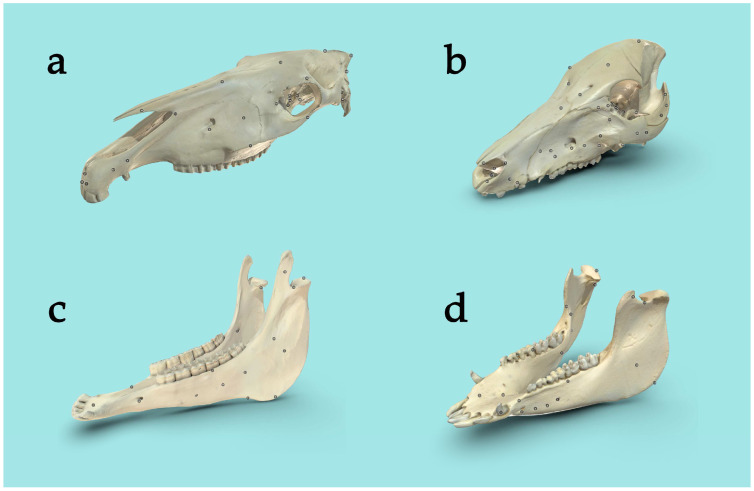
Annotated 3D scans of the horse’s upper skull (**a**) and mandible (**c**), as well as the pig’s upper skull (**b**) and mandible (**d**).

Once the 3D scans were finished, they were exported from Artec Studio 16 and uploaded and annotated on Sketchfab (Figure 1), a 3D modeling platform website where 3D content can be shared, viewed and annotated (www.sketchfab.com; Sketchfab, New York, NY, USA). The 3D scans can be utilized on most technical devices with internet access. Within Sketchfab, the 3D objects can be zoomed in, zoomed out, and rotated in all 3D axes. On the horse skull, 68 anatomical structures and on the pig skull 71 structures were annotated (Supplement S2). With a mouse click or tap on a specific structure of the 3D scan, the respective annotations get presented to the users. 

#### 2.2.3. 2D Images

**Figure 2:**
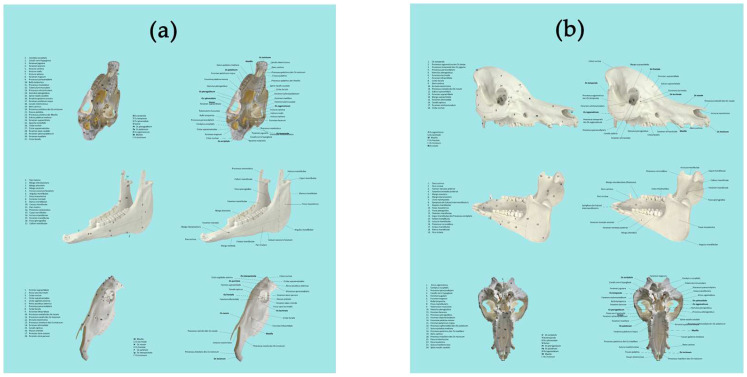
(**a**) 2D images of the horse skull, annotated with numbers (left) or with arrows (right), (**b**) 2D images of the pig skull, annotated with numbers (left) or with arrows (right).

2D images of the horse and pig skulls were created using a system camera (Sony, Alpha NEX 3, Nihombashi, Tokio, Japan) and an iPhone (Apple Inc., 13. Generation, One Apple Park Way, Cupertino, CA, USA). The pictures were edited and annotated using PowerPoint (Office PowerPoint 2019, Windows). Identical structures were either portrayed using numbers (2D numbers) or arrows (2D arrows). The structures shown were exactly alike the ones annotated in the 3D scans (Figure 2). A close-up depiction of 3D and 2D annotations of selected structures can be found in Supplement S3. 

### 2.3. Tests

#### 2.3.1. Mental Rotation Test

The mental rotation test was used to assess the students’ spatial ability [20]. The test had a time limit of 10 min and consisted of 18 exercises showing pictures of diverse tube figures placed in a transparent cube. Each exercise comprises two pictures of a cube, with the left one showing the cube in an initial position and the right one showing the cube from a different viewpoint. The objective of this test is to determine the position from which one observes the rotated box (right, left, up, down, behind) [20].

#### 2.3.2. Knowledge Tests

**Figure 3:**
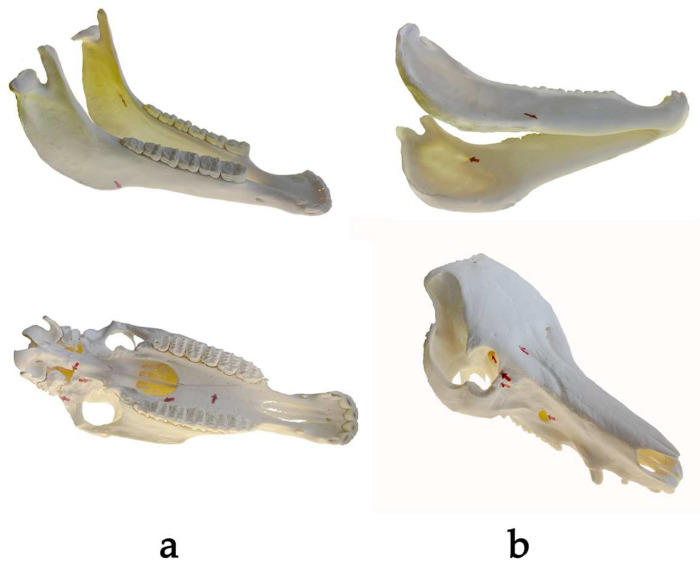
Annotated skulls of the horse (**a**) and of the pig (**b**) using adhesive arrows.

Regarding each knowledge test, which was conducted before (pre-test) and after (post-test) the learning period, 10 structures were annotated with adhesive arrows. Each arrow was marked with a certain number and was attached to the horse and pig skulls, respectively (Figure 3). In each skull, 5 structures were identical in the pre- and post-test, while the other 5 structures were replaced by others in the post-test. During study 1, one tag got lost on pig skulls during the changeover process, resulting in one less queried structure in the post-test in comparison to the horse skull. The students’ tasks in both knowledge tests consisted of writing down the anatomical terms of the annotated structures behind the corresponding numbers on the examination sheets (Supplement S4). 

### 2.4. Student Evaluation 

After performing study 1 and study 2, respectively, all students got access to all learning materials to prepare for the regular exam. After the exam, participants were requested to fill out a brief questionnaire regarding their experience of the study process. The evaluation was carried out with LimeSurvey (LimeSurvey, Version 5.6.31).

For the evaluation of the learning tools, responses for the first five questions were registered for each statement using a six-point Likert scale (1 = strongly disagree, 6 = strongly agree). Question six was a ranking, and the last three questions were noted as free-text responses. Questions and response types are listed in Table 1.

### 2.5. Study Design

Study 1 and study 2 were conducted during regular anatomy lessons on the skull, in which students were regularly divided into 32 groups, each group being located at one dissection table and consisting of 5 to 6 students. Before the lesson began, 16 groups were randomly assigned to the study group 3D scans and the other 16 groups for 2D images. The study group of 2D images was further divided equally into 2D arrows and 2D numbers.

All tests were conducted by each student individually while learning time was spent in the respective groups, in which the students were allowed to interact with each other. The study began with the mental rotation test. The test started for all students at the same time. After the time had elapsed, all students stopped simultaneously. For the subsequent pre-test, annotated skulls were covered with blankets. After the students had entered their personal code and selected whether they belonged to the 3D or 2D group on their test sheet, all students began the knowledge test at the same time by removing the blankets from the skulls. At the end of the test period, the students flipped their test sheets upside down on the table. Subsequently, the students began their learning period on the non-annotated skulls of horses and pigs. In the 3D scans group, each student received the annotated 3D scans of the skulls on an iPad from Apple (Apple Inc., One Apple Park Way, Cupertino, CA, USA), of the 9th generation in study 1 and the 10th generation in study 2. In the 2D image groups, each student received a set of color-printed annotated images of the skulls. 

After the learning time, the post-test proceeded identically to the pre-test, including filling out the personal code on the examination sheet again and selecting their study group (3D scans, 2D arrows, or 2D numbers). The schedule of study 1 and study 2 is shown in Table 2.

### 2.6. Statistical Analysis

Statistical and descriptive analyses and charts were carried out with IBM SPSS Statistics Version 29^®^ (IBM, Armonk, New York, NY, USA). Only the data from people who submitted all three test forms were regarded for analysis. In order to preserve the anonymity of the participants, but to merge the three tests (pre-test, post-test, mental rotation test) of the respective person, an individual code of five letters was used, consisting of the third letter of the month of birth of their mother, the third letter of their month of birth, the second letter of their mother’s first name, the second letter of their first name, and the second letter of their birthplace. In addition, the students indicated in the post-test which study group they were assigned to. In study 2, four students had to be excluded due to not submitting the three required forms and one student had to be excluded due to missing indication of the study group. 

The mental rotation test was a single-choice assessment, with one point awarded for each correct answer. Incorrect or missing answers received no points. A descriptive analysis of data was conducted. Based on the random distribution of students to the study groups, no differences between the study groups were expected. 

In the knowledge test, each correct term was awarded one point. No points were given for incorrect or missing terms. If terms were misspelled or incomplete, an assessment was made as to whether the term written down indicated that the correct structure had been recognized. To determine whether an individual’s assessment is sufficient, the pre- and post-tests were evaluated by two researchers. Due to the minimal deviation (less than 10% in both studies), the assessment from one researcher was considered sufficient for the analysis.

The learning outcome was measured by calculating the difference of sums between post-test and pre-test. The mean score difference between the pre-test and post-test was then calculated for all study groups and compared using a paired *t*-test, followed by the analysis of variance (ANOVA) to determine the differences between study groups. Pearson’s correlation was utilized to assess the correlation between spatial ability and learning outcomes of each study group of study 1 and study 2 separately.

Analysis of variance was performed to test the influence of the mental rotation test, the study groups, and study 1 and study 2 including the interaction term on the mean score difference. Levene’s test revealed that the variances between the study groups in study 2 were not equal, and thus, robust parameter estimates were assessed. The normality and homoscedasticity of residuals were inspected visually. Raw data can be found in Supplement S5.

## 3. Results

### 3.1. Number of Participants 

A total of 278 second-year veterinary medicine students participated in total in study 1 and study 2. They were distributed as follows: In study 1, 118 students were involved, among them 59 students joined the study group with 3D scans, 33 students with 2D numbers, and 26 students with 2D arrows. Study 2 included 160 students, 79 students joined the study group with 3D scans, 39 students with 2D numbers, and 42 students with 2D arrows. 

### 3.2. Spatial Ability 

**Figure 4:**
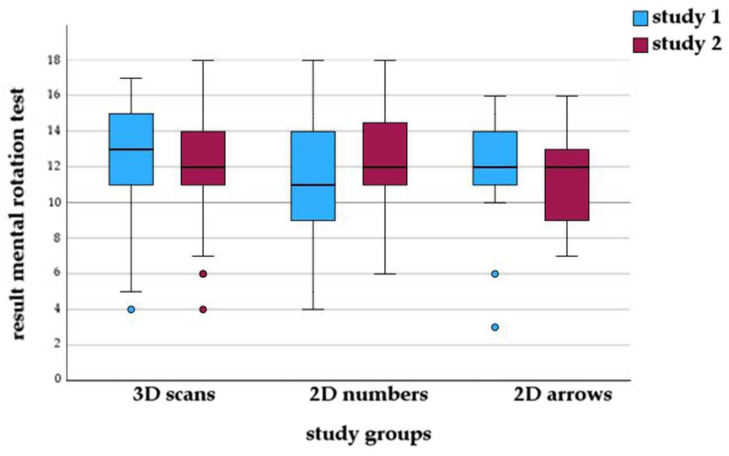
Boxplot of mental rotation test scores in the groups with different learning materials (annotated 3D scans, 2D images annotated with numbers or arrows) in study 1 and study 2.

As displayed in Figure 4, the distribution of the mental rotation test results was similar in all groups (study 1: *p* = 0.195, study 2: *p* = 0.308).

### 3.3. Learning Outcome 

Regarding the post-test, students reached significantly higher score points than in the pre-test (mean pre-test = 0.86, mean post-test = 8.3, *p* < 0.001). The post-test values were higher in study 1 (11.4 ± 4.2) than in study 2 (6.0 ± 3.1). 

**Figure 5:**
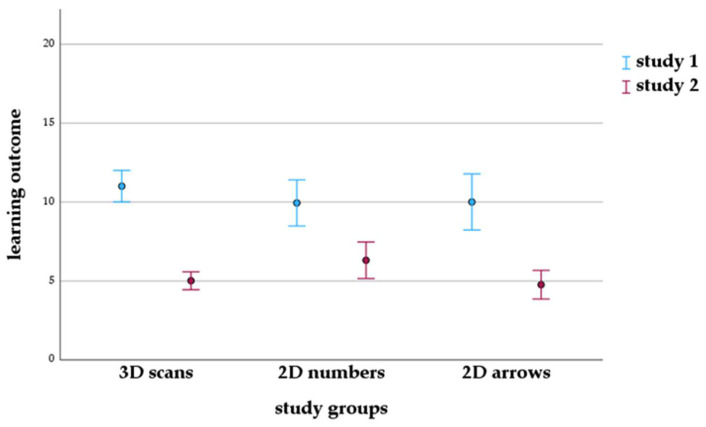
Estimated means and 95% confidence intervals of learning outcome in the groups with different learning materials (annotated 3D scans, 2D images annotated with numbers or arrows) in study 1 and study 2.

Analysis of variance revealed that the average learning outcome in study 1 was 5.2 score points higher than in study 2 (*p* < 0.001) and that there was an interaction between study 1 and study 2 and the study groups (*p* = 0.121, Figure 5). In study 1, the highest learning outcome was achieved by the 3D scan group, and in study 2 by the 2D numbers group (Figure 5). However, study groups did not differ significantly in their mean learning outcome (*p* = 0.500). In study 1, mean learning outcome values were 11.0 ± 3.8 for the 3D scans group and 9.97 ± 4.21 for the 2D images group (*p* = 0.165). A post hoc power analysis (conducted with G*Power 3.1.9.2) showed a medium effect (d = 0.57) and a power of 86%. Under these conditions, a significant difference between the two groups would have required a sample size of 83 students in each group. Even by comparing 2D arrows (10.0 ± 4.4) and 2D numbers (9.94 ± 4.1) to the 3D scan group, no significant difference was shown (*p* = 0.383). In study 2, mean learning outcomes were 5.0 ± 2.5 for the 3D scans group and 5.51 ± 3.32 for the 2D images group (*p* = 0.292). A post hoc power analysis revealed no effect (d = 0.00) and a power of 5%. However, when comparing 2D numbers (6.31 ± 3.6) and 2D arrows (4.76 ± 2.9) to the 3D scan group, the 2D numbers group was significantly higher (*p* = 0.035).

### 3.4. Correlation of Spatial Ability and Learning Outcome 

**Figure 6:**
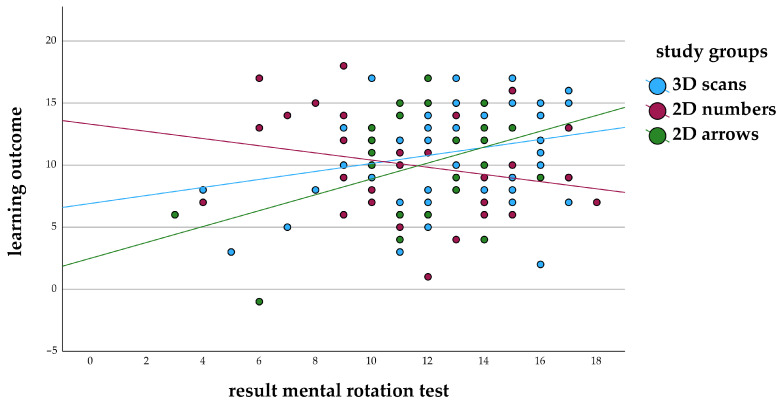
Correlation of learning outcome and results of the mental rotation test in the groups with different learning materials (annotated 3D scans, 2D images annotated with numbers or arrows) in study 1.

**Figure 7:**
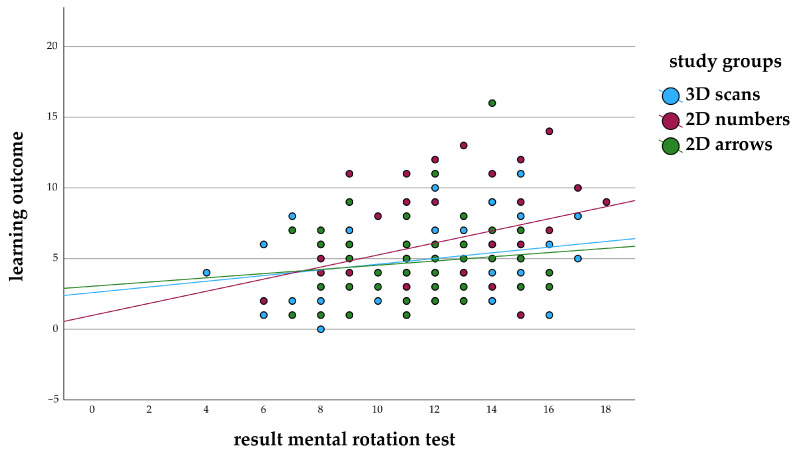
Correlation of learning outcome and mental rotation test in the groups with different learning materials (annotated 3D scans, 2D images annotated with numbers or arrows) in study 2.

The correlation between learning outcome and spatial ability, measured by the mental rotation test, was positive (*p* = 0.160) in study 1 for the study group of 3D scans with only slight differences in the correlation between study 1 (r = 0.244) and study 2 (r = 0.223). 2D numbers showed a negative correlation coefficient in study 1 (r = –0.245) (Figure 6) and a positive correlation coefficient in study 2 (r = 0.309) (Figure 7). A positive correlation was also found for 2D arrows in both studies (study 1: r = 0.400; study 2: r = 0.128).

### 3.5. Student Evaluation

The questionnaire to evaluate the learning tools was completed by 130 students after study 1 and 144 students after study 2. Responses were analyzed using a six-point Likert scale (1 = strongly disagree, 6 = strongly agree). Results in the range of 4 to 6 were summed up and defined as “agreeing to this statement”, as well as the sums of the answers of 1 to 3 representing “disagreeing with this statement”. In both studies, most of the students agreed on feeling well prepared for a practical exam by using 3D scans (74.62% in study 1, 71.09% in study 2). 58.46% in study 1 and 57.81% in study 2 agreed on feeling well prepared for an exam using pictures with arrows. By learning with pictures with numbers, 53.08% in study 1 and 46.87% in study 2 agreed on feeling well prepared for an exam. 53.84% in study 1 and 46.83% in study 2 agreed on 3D scan controls (turn, rotate, zoom) being easy to handle.

Afterward, the students were asked to indicate their order of preference for learning materials. In study 1, students most frequently chose 3D scans in first place (52.31% 3D scans, 20% 2D arrows, 13.08% 2D numbers), 2D arrows in second place (34.62% 2D arrows, 28.46% 2D numbers, 22.31% 3D scans) and 2D numbers in third place (43.08% 2D numbers, 30.77% 2D arrows, 10.77% 3D scans). In study 2, students also most frequently placed 3D scans in first place (61.06% 3D scans, 24.78% 2D arrows, 14.16% 2D numbers), 2D arrows in second place (41.44% 2D arrows, 30.63% 2D numbers, 27.93% 3D scans) and 2D numbers in third place (54.95% 2D numbers, 33.33% 2D arrows, 11.71% 3D scans). 

Finally, participants were asked about the negative and positive aspects they noticed while using the visual teaching materials. A few negative aspects were mentioned, mostly referring to technical issues and difficulties in handling and orientating while using 3D scans, e.g., “slight difficulties to rotate and zoom with the 3D scans”; “The computer did not always keep up. It got hot and the pictures were slow”; and, “When you tap on an annotation, the image sometimes shifts and you are in a different place than you want to be”. The mentioned positive aspects referred to a good visualization of structures, learning benefits, and availability of scans, e.g., “The visualization of the 3D scans worked well. I could visualize the skull well in my hand”; “With the 3D scans, I can memorize it faster and my attention span is greater”; and, “It is good to have the anatomical object available at home, too, which is good for learning and self-monitoring”.

For 2D images, some negative comments were mentioned about the annotation with arrows being confusing, e.g., “It was not clear which structure was mentioned” and “The structures are easier to recognize in 3D scans than in 2D images”. However, some positive aspects of the 2D images were referring to 2D numbers giving a good overview of the picture’s structures “They were good to quiz yourself”.

## 4. Discussion

In the presented study, the effectiveness of 3D scans versus 2D images as learning materials in veterinary anatomy classes was investigated. This research was based on the hypothesis that students using 3D scans for learning anatomical structures of skulls would achieve a higher learning outcome compared to those using 2D images. Moreover, the relation between spatial ability and the type of learning material was examined, based on the hypothesis that students with low spatial ability achieve greater success using 3D scans than 2D images.

Analysis of the data indicates that there is a significant difference between pre-test and post-test of the study groups 2D images as well as for 3D scans. This leads to the conclusion that all study groups in study 1 and study 2 were able to identify more anatomical structures correctly after the learning period. However, when comparing the study groups, no significant difference between 2D images and 3D scans was detectable. Similar findings were observed in other studies conducted to compare the effectiveness of 2D and 3D methods in anatomy education. One study tested the use of a digital 3D skull model in anatomy teaching. It was shown that the skull virtual learning resource was equally efficient as the cadaver skull and 2D atlas in teaching anatomy structures [21]. Moreover, a study by the University of Bristol evaluated a 3D computer model of the equine paranasal sinuses as a tool for veterinary anatomy education comparing it to 2D-based visualizations in a pre-test/post-test design. Also in this research, no statistically significant difference was found between 3D and 2D groups [22]. In contrast, a previous study pointed out that dynamic visualizations, including 3D models, can greatly enhance students’ understanding of complex concepts in subjects such as geology, biology, and chemistry. However, the impact varied significantly between different groups of students, suggesting that factors like prior knowledge and learning styles could influence the effectiveness of these tools [23]. Thus, the hypothesis that students who use 3D scans as learning material will achieve higher learning outcomes compared to those who use 2D images could not be supported.

Moreover, time as a factor plays a crucial role in the study. In study 1, students had 120 min of learning time for both skulls. This amount seemed to be more than necessary, which was shown by students already leaving the dissection hall before time ran out. This led to the performance of study 2 which involved a shorter learning time of 40 min for learning all structures of both skulls. Since the students in study 2 had a significantly lower learning outcome, it can be assumed that this learning time was too short. Assuming that the learning time in study 1 was too long and that the average learning outcome of study 1 was, therefore, the maximum achievable, a calculated learning time of approx. 75 min would be sufficient, assuming that learning takes place in a linear fashion. In study 1, the study group 3D scans displayed the tendency of being the group with the highest learning outcome. In contrast, in study 2, which was conducted under time pressure, it was shown that 2D numbers had a significantly better learning outcome than the other groups. These findings indicate that having more time to learn a new tool makes a difference in the learning outcome, especially if it is a completely new tool that has never or occasionally been used by the participants [24,25,26,27] Students are used to learning with 2D images since school and rarely get in touch with 3D models [28]. Although students are increasingly using tablet computers as a learning tool in the dissection courses, they still mainly use them to access 2D-based visualizations, as books or their own scripts and no 3D models. It takes time to understand how to interact with the software of Sketchfab and get to know how to use the 3D scans [29]. This was mentioned by some participants as a negative aspect of the evaluation. 

However, the high learning outcome for 2D numbers might be based on the regular usage of this certain type of annotation. Various veterinary anatomy book images are annotated with numbers. Therefore, veterinary medicine students are used to learning with this learning material. A former study stated that annotations, particularly numbered ones, significantly helped undergraduate students engage with scientific texts more deeply [30]. These annotations acted as frameworks, making complex information more accessible and enhancing students’ comprehension and retention. Positive student feedback indicated that these annotations were especially useful for understanding graphs and interpreting scientific vocabulary, highlighting the effectiveness of numbered annotations in educational contexts [30]. Another study investigated the role of annotations in the comprehension of scientific texts, focusing on cognitive load effects. The study found that well-structured annotations, such as numbered lists, helped reduce cognitive load and improved comprehension, indicating that numbered annotations can provide clear and organized information, facilitating better understanding and learning outcomes [31]. 

Several findings indicate that students with low spatial ability would have a higher learning outcome using 3D scans than 2D images [16,19]. In study 1, the analysis of the correlation between learning outcome and mental rotation test showed that students on the lower scale of mental rotation test results had higher learning outcome values using 3D scans than 2D arrows. However, the same correlation was not reproduced in study 2. Therefore, the hypothesis that the 3D scans support students with weaker spatial ability could not be supported.

In the student evaluation, 3D scans were chosen as the preferred learning material, so they might enhance students’ motivation. Another study showed that using 3D scans improved participants’ motivation levels when compared with 2D learning materials [22,32]. Therefore, even if 3D scans do not directly result in improved learning outcomes, the element of pleasure remains crucial to the learning process [32]. It plays a significant role in motivating students, driving them to actively engage with the topic and ultimately achieve their educational goals [33]. Innovative learning methods can significantly enhance student motivation and engagement, leading to improved knowledge retention and learning outcomes [34,35,36]. Engaging teaching approaches can indirectly boost learning outcomes by increasing overall motivation, improving attendance, and inspiring students to adopt novel methods in their self-directed learning [37]. Additionally, the evaluation of the learning tools revealed some limitations associated with the 3D scans. These addressed issues primarily involved the use, handling, and certain technical restrictions of the 3D scans. Among the most significant challenges were the annotations created on Sketchfab. These annotations were found to be less user-friendly due to their tendency to rotate quickly and zoom in excessively, which made them difficult to navigate and interact with effectively, potentially harming the process of capturing the 3D shape. Furthermore, unlike the 2D images, Sketchfab does not provide an overview of all the annotations on the respective skull. Instead, the annotations can only be opened one at a time, which can further lead to limiting the learning process. Finding the ideal annotations for 3D models can often prove to be a challenging task [38,39]. Another limitation inherent in using 3D scans is the requirement for compatible technical devices capable of adequately processing and displaying the 3D scans. Consequently, the utilization of 3D scans necessitates the availability of suitable technology to ensure optimal viewing and utilization. The inequality of access to online technology and devices and the possibly unreliable internet makes the 3D scans not accessible to everyone anytime [40]. Interestingly, the evaluation showed that the students favored annotation by arrows over annotation by numbers for 2D images, although in study 2, under time pressure, the 2D numbers group achieved the best learning outcome. This finding confirms indications that the learning materials preferred by the students do not necessarily lead to the highest learning outcome for them [41].

One of the limitations of this study could be that the studies only had one assessment time-point, taking place during a dissection course. Additionally, these studies faced technological constraints, as they were unable to test a wide range of devices for displaying the 3D scans. This limitation has resulted in findings that are specific to the particular equipment used. Another limitation is that conclusions about the efficacy of 3D scans cannot be drawn to other anatomical structures based on the exclusive use of 3D scans of the skull. 

Future research could involve conducting a comprehensive comparison among various 3D platforms, focusing particularly on their user-friendliness concerning both annotating 3D models and accessing them technically. This comparative analysis could explore interface design, ease of navigation, annotation tools, and technical requirements, aiming to provide valuable insights into optimizing the usability and accessibility of 3D platforms for educational and professional purposes. In order to eradicate the limitation of raising data solely at one assessment time-point and to analyze long-term retention of anatomical knowledge by using 3D scans, a reassessment of students’ learning outcomes should be conducted involving multiple time points. To assess conclusions on other anatomical structures, similar studies could be conducted using 3D scans of different organs. Ultimately, conducting a study where students have the opportunity to familiarize themselves with the 3D surface before being introduced to the skulls would be beneficial, as it allows them to analyze learning success with the starting conditions being more aligned. 

## 5. Conclusions

This study did not provide significant support for the hypothesis that students who use 3D scans to learn anatomical structures have a higher learning outcome compared to students who use 2D images. It was found that 3D scans do not lead to superior learning outcomes compared to 2D-based learning materials. Similarly, the hypothesis that students with low spatial ability have a higher learning outcome with the use of 3D scans than with 2D images is also not confirmed. Students with low spatial ability did not experience significantly better learning outcomes with the use of 3D scans. However, the data does provide valuable insights into the role of 3D emerging tools in veterinary anatomy education. Although practicing the handling of 3D scans appears to be time-consuming, working with 3D scans seems to enhance student motivation. The reduced learning time may have had a bigger impact on the 3D group, based on the introduction of novel learning materials, which required additional time to be understood and effectively integrated with the learning process. Similarly, the findings of this study highlight the crucial importance of allowing students adequate time to adapt to new circumstances. This need is evident not only in the specific context of the 3D skull but also more broadly in the first semester; it was noted, for example, that, expectations, relationships with professors, and the nature of examinations are significantly different than the school environment. The results of the current study provide evidence that supports the educational effectiveness of 3D scans as a complementary method to understanding anatomical structures.

## Figures and Tables

**Table 1:** Question and response types of the student evaluation.

Question	Response Type
1. By learning with the 3D scans, I felt well prepared for a practical exam on an anatomical specimen.	Likert scale
2. By learning with the pictures with arrows, I felt well prepared for a practical exam on an anatomical specimen.	Likert scale
3. By learning the pictures with numbers, I felt well-prepared for a practical exam on an anatomical specimen.	Likert scale
4. The controls (turn, rotate, zoom) of the 3D scans are easy to handle.	Likert scale
5. The controls (turn, rotate, zoom) of the 3D scans are intuitive.	Likert scale
6. Please indicate the order of your preference for these learning materials (3D scans, 2D arrows, 2D numbers).	Ranking
7. What positive aspects did you notice while using the visual teaching materials (3D scans, labeled specimen images)?	Free text
8. What negative aspects did you notice when using the visual teaching materials (3D scans, labeled specimen images)?	Free text
9. Do you have any feedback for us regarding the course of the study?	Free text

**Table 2:** Schedule of study 1 and study 2.

Mental rotation test	10 min (study 1 and study 2)
Knowledge test (pre-test)	15 min (study 1 and study 2)
Learning period	120 min (study 1), 40 min (study 2)
Knowledge test (post-test)	15 min (study 1 and study 2)

## Data Availability

Data are contained within the article or Supplementary Material. The data presented in this study are available in the Supplementary Material.

## Supplementary Materials

The following supporting information can be downloaded at: https://www.mdpi.com/article/10.3390/ani14152171/s1, Supplement S1: Information letter and data protection declaration, Supplement S2: List of annotated structures, Supplement S3: Close-up depiction of 3D and 2D annotations, Supplement S4: Examination sheets, Supplement S5: Raw data.
