# Supplementary material for: Effects of 3D Scans on Veterinary Students’ Learning Outcomes Compared to Traditional 2D Images in Anatomy Classes
Source: Animals (Basel). 2024 Jul 25;14(15):2171. doi: 10.3390/ani14152171 (PMC11311044; doi:10.3390/ani14152171)
Supplement: Supplementary file 1 [file animals-14-02171-s001.zip › Supplement S1.pdf]

"Investigation of the didactic potential of 3D scans in veterinary anatomy teaching"

Dear students of veterinary medicine,

Thank you for agreeing to participate in the study "Investigation of the didactic potential of 3D scans in veterinary anatomy teaching". We would like to inform you in advance about the study, the procedure and some data protection aspects. Please read the following information carefully.

OBJECTIVE OF THE STUDY: This study investigates the potential added value of annotated 3D scans as an information and learning tool in veterinary anatomy teaching. The study is purely for scientific purposes and to improve the quality of teaching and is part of a dissertation project.

WHAT YOUR PARTICIPATION CONSIST OF: Your participation consists of taking part in a short knowledge test to assess your prior knowledge and a test of spatial ability in anonymized form. You will then receive either annotated images (control group) or annotated 3D scans (test group) on an iPad of the specimens regularly covered in the anatomy course. In order to be able to measure the learning outcome, you will also be given an anonymized knowledge test at the end of the study and a voluntary evaluation of the learning materials at a later date.

TIME FRAMEWORK/EXPENSES: The study will be carried out as part of the anatomy course using the topics and specimens regularly covered in the course, so that you will not need to invest any additional time or learning effort.

RISKS: Following the study, all learning materials will be made available to all students, so that you will not suffer any disadvantages regardless of whether you will be in the control or test group.

BENEFITS: Participation in the study offers you the opportunity to contribute to the development of new and innovative teaching methods in veterinary anatomy.

CONFIDENTIALITY AND DATA PROTECTION: All data will be collected anonymously. You will be asked to generate an individual code in order to be able to associate the initial and final knowledge test with each other. The consent forms are stored in a separate folder in a secure location that is only accessible to the project management. The data will be protected in accordance with the strictest data protection regulations in accordance with the European General Data Protection Regulation (GDPR), which came into force on 25 May 2018. The survey data will only be processed by the persons involved in the project and used exclusively for the purposes of research and quality assurance of studies and teaching.

CONSENT TO PARTICIPATE AND WITHDRAWAL: Participation in the study is completely voluntary and you will not suffer any disadvantages if you do not participate. You have the option of withdrawing from the study at any time by informing us that you no longer wish to participate in the study. This will also not result in any disadvantages for you and no questions will be asked about the reasons for your withdrawal.

Data protection declaration of consent: I hereby declare that I have carefully read and understood the above information and explanations. I give my consent to the collection, storage, processing and use of my data solely for the purpose of answering scientific questions in connection with this study.

____

First name, last name Date Signature
