# Supplementary material for: Effects of 3D Scans on Veterinary Students’ Learning Outcomes Compared to Traditional 2D Images in Anatomy Classes
Source: Animals (Basel). 2024 Jul 25;14(15):2171. doi: 10.3390/ani14152171 (PMC11311044; doi:10.3390/ani14152171)
Supplement: Supplementary file 1 [file animals-14-02171-s001.zip › Supplement S2.pdf]

List of annotated structures

Horse skull

Upper skull:

1. Os incisivum
2. Canalis interincisivus
3. Processus alveolaris des Os incisivum
4. Processus nasalis des Os incisivum
5. Processus palatinus des Os incisivum
6. Maxilla
7. Crista facialis
8. Foramen infraorbitale
9. Os nasale
10. Processus rostralis des Os nasale
11. Incisura nasoincisiva
12. Os frontale
13. Foramen supraorbitale
14. Os zygomaticum
15. Fossa sacci lacrimalis
16. Foramen sphenopalatinum
17. Foramen maxillare
18. Os sphenoidale
19. Foramen alare caudale
20. Tuberculum musculare
21. Foramen lacerum
22. Incisura carotica
23. Incisura ovalis
24. Incisura spinosa
25. Foramen jugulare
26. Canalis nervi hypoglossi
27. Processus paracondylaris
28. Condylus occipitalis
29. Bulla tympanica
30. Processus mastoideus
31. Porus acusticus externus
32. Crista sagittalis externa
33. Crista nuchae
34. Protuberantia occipitalis externa
35. Foramen magnum
36. Foramen ethmoidale
37. Canalis opticus
38. Fissura orbitalis
39. Foramen alare rostrale
40. Foramen alare parvum
41. Processus retroarticularis

42. Hamulus pterygoideus
43. Spina nasalis caudalis
44. Foramina palatina minora
45. Foramen palatinum majus
46. Dens caninus
47. Fissure palatina
48. Sutura palatina mediana
49. Crista supramastoidea
50. Processsus palatinus der Maxilla

Mandible:

51. Pars incisiva
52. Margo interalveolaris
53. Margo alveolaris
54. Margo ventralis
55. Incisura vasorum facialium
56. Angulus mandibulae
57. Fossa masseterica
58. Foramen mentale
59. Ramus mandibulae
60. Corpus mandibulae
61. Pars molaris
62. Processus coronoideus
63. Caput mandibulae
64. Incisura mandibulae
65. Foramen mandibulae
66. Fossa pterygoidea
67. Collum mandibulae
68. Linea mylohyoidea

Pig skull

Upper skull:

1. Processus zygomaticus des Os temporale
2. Processus temporalis des Os zygomaticum
3. Processus paracondylaris
4. Hamulus pterygoideus
5. Foramina lacrimalia
6. Foramen infraorbitale
7. Crista facialis
8. Dens caninus
9. Incisura nasoincisiva
10. Processus rostralis des Os nasale
11. Sulcus supraorbitalis
12. Foramen supraorbitale
13. Margo supraorbitalis
14. Foramen ethmoidale

15. Canalis opticus
16. Foramen orbitorotundum
17. Crista nuchae
18. Os incisivum
19. Os nasale
20. Os frontale
21. Fossa sacci lacrimalis
22. Os occipitale
23. Condylus occipitalis
24. Tuberculum musculare
25. Bulla tympanica
26. Foramen lacerum
27. Foramen jugulare
28. Canalis nervi hypoglossi
29. Processus pterygoideus
30. Foramina palatina minora
31. Foramen palatinum majus
32. Maxilla
33. Sutura palatina mediana
34. Processus sphenoidalis des Os palatinum
35. Fossa mandibularis
36. Foramen stylomastoideum
37. Foramen magnum
38. Porus acusticus externus
39. Os temporale
40. Os lacrimale
41. Processus palatinus des Os maxillare
42. Processus maxillaris des Os incisivum
43. Fissura interincisiva
44. Fissura palatina
45. Sutura maxilloincisiva
46. Pina nasalis caudalis
47. Foramen sphenopalatinum
48. Foramen maxillare
49. Os zygomaticum
50. Arcus zygomaticus

Mandible:

51. Dens caninus
52. Pars incisiva
53. Foramina mentalia
54. Foramina mentalia
55. Formina mentalia
56. Formina mentalia
57. Margo alveolaris
58. Margo interalveolaris
59. Linea mylohyoidea

60. Symphysis (et Sutura) intermandibularis
61. Angulus mandibulae
62. Fossa masseterica
63. Fossa pterygoidea
64. Foramen mandibulae
65. Caput mandibulae
66. Collum mandibulae
67. Incisura mandibulae
68. Processus coronoideus
69. Corpus mandibulae
70. Ramus mandibulae
71. Pars molaris
