# Supplementary material for: Effects of 3D Scans on Veterinary Students’ Learning Outcomes Compared to Traditional 2D Images in Anatomy Classes
Source: Animals (Basel). 2024 Jul 25;14(15):2171. doi: 10.3390/ani14152171 (PMC11311044; doi:10.3390/ani14152171)
Supplement: Supplementary file 1 [file animals-14-02171-s001.zip › Supplement S4.pdf]

Please select: **3D scans group** ☐ **2D images group** ☐

Personal code:

Third letter of your mother's birth month	
Third letter of your birth month	
Second letter of your mother's first name	
Second letter of your first name	
Second letter of your place of birth	

Note: If unknown, please enter an O

Pre-test

	**Horse**
1	*Correct answer: Foramen ethmoidale*
2	*Correct answer: Foramen mandibulae*
3	*Correct answer: Foramen alare caudale*
4	*Correct answer: Processus retroarticularis*
5	*Correct answer: Incisura carotica*
6	*Correct answer: Foramen maxillare*
7	*Correct answer: Tuberculum musculare*
8	*Correct answer: Fossa pterygoidea*
9	*Correct answer: Canalis interincisivus*
10	*Correct answer: Canalis nervi hypoglossi*
	**Pig**
11	*Correct answer: Foramen orbitorotundum*
12	*Correct answer: Foramen supraorbitale*
13	*Correct answer: Linea mylohyoidea*
14	*Correct answer: Processus pterygoideus*
15	*Correct answer: Foramina lacrimalia*
16	*Correct answer: Foramina mentalia*
17	*Correct answer: Sutura maxilloincisiva*
18	*Correct answer: Fissura palatina*
19	*Correct answer: Hamulus pterygoideus*
20	*Correct answer: Canalis opticus*

Please select: **3D scans group** ☐ **2D arrows group** ☐ **2D numbers group** ☐

Personal code:

Third letter of your mother's birth month	
Third letter of your birth month	
Second letter of your mother's first name	
Second letter of your first name	
Second letter of your place of birth	

Note: If unknown, please enter an O

Post-test

	**Horse**
1	*Correct answer: Foramen ethmoidale*
2	*Correct answer: Foramen mandibulae*
3	*Correct answer: Foramen alare caudale*
4	*Correct answer: Processus retroarticularis*
5	*Correct answer: Incisura carotica*
6	*Correct answer: Foramen alare parvum*
7	*Correct answer: Foramen palatinum majus*
8	*Correct answer: Sutura palatina mediana*
9	*Correct answer: Incisura vasorum facialium*
10	*Correct answer: Foramen sphenopalatinum*
	**Pig**
11	*Correct answer: Foramen orbitorotundum*
12	*Correct answer: Foramen supraorbitale*
13	*Correct answer: Linea mylohyoidea*
14	*Correct answer: Processus pterygoideus*
15	*Correct answer: Foramina lacrimalia*
16	*Correct answer: Foramen stylomastoideum*
17	*Correct answer: Foramina palatina minora*
18	*Correct answer: Porus acusticus externus*
19	*Correct answer: Foramen jugulare*
20	*Correct answer: Foramen infraorbitale*
