# Supplementary figures and images for: Effects of 3D Scans on Veterinary Students’ Learning Outcomes Compared to Traditional 2D Images in Anatomy Classes
Source: Animals (Basel). 2024 Jul 25;14(15):2171. doi: 10.3390/ani14152171 (PMC11311044; doi:10.3390/ani14152171)

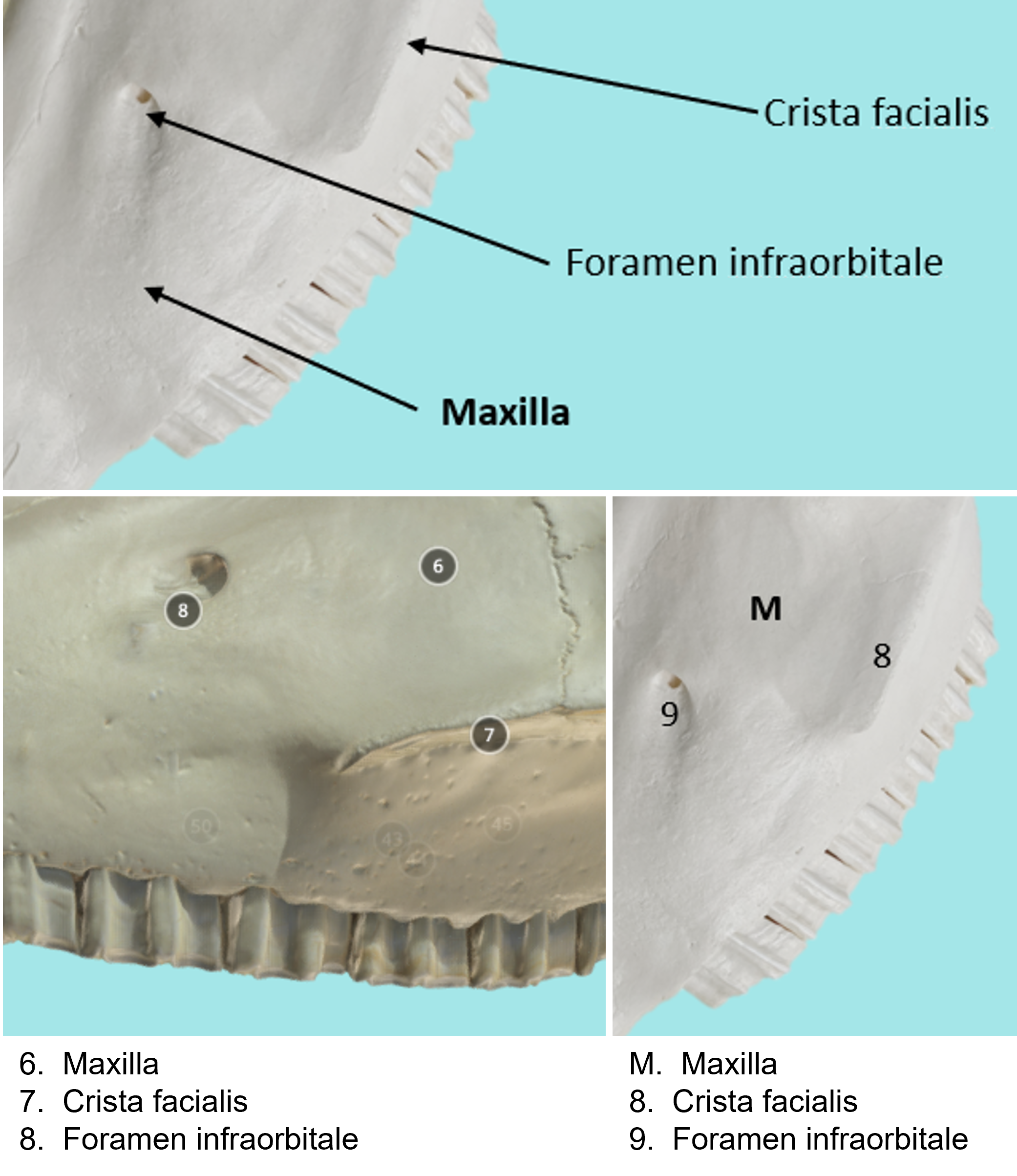

Supplement: Supplementary file 1 [file animals-14-02171-s001.zip › Supplement S3.png]
